# Real-world evaluation of MRI fistula volume as a radiological biomarker of disease activity in perianal fistulizing Crohn’s disease

**DOI:** 10.1093/ecco-jcc/jjaf216

**Published:** 2025-11-29

**Authors:** Easan Anand, Sulak Anandabaskaran, Theo Pelly, Ailsa Hart, Phil Tozer, Phillip Lung

**Affiliations:** St Mark’s, The National Bowel Hospital, Central Middlesex, Acton Lane, London NW10 7NS, United Kingdom; Department of Surgery & Cancer, Imperial College London, Exhibition Rd, South Kensington, London SW7 2AZ, United Kingdom; St Mark’s, The National Bowel Hospital, Central Middlesex, Acton Lane, London NW10 7NS, United Kingdom; St Mark’s, The National Bowel Hospital, Central Middlesex, Acton Lane, London NW10 7NS, United Kingdom; Department of Surgery & Cancer, Imperial College London, Exhibition Rd, South Kensington, London SW7 2AZ, United Kingdom; St Mark’s, The National Bowel Hospital, Central Middlesex, Acton Lane, London NW10 7NS, United Kingdom; Department of Metabolism, Digestion & Reproduction, Imperial College London, Exhibition Rd, South Kensington, London SW7 2AZ, United Kingdom; St Mark’s, The National Bowel Hospital, Central Middlesex, Acton Lane, London NW10 7NS, United Kingdom; Department of Surgery & Cancer, Imperial College London, Exhibition Rd, South Kensington, London SW7 2AZ, United Kingdom; St Mark’s, The National Bowel Hospital, Central Middlesex, Acton Lane, London NW10 7NS, United Kingdom; Department of Surgery & Cancer, Imperial College London, Exhibition Rd, South Kensington, London SW7 2AZ, United Kingdom

**Keywords:** pfCD, TOpClass classification, MRI fistula volume, radiological healing, radiological improvement

## Abstract

**Background:**

The TOpClass classification for perianal fistulizing Crohn’s disease (pfCD) facilitates tailored treatment to clinical state and patient goals. MRI is central to pfCD assessment, but existing indices are limited in predicting disease classification and trajectory. This study evaluated MRI fistula volume as a radiological biomarker and its longitudinal association with pfCD class.

**Methods:**

We conducted a retrospective cohort study of 51 consecutive pfCD patients who underwent a baseline pelvic MRI in 2010 with ≥1 follow-up MRI. pfCD class was assigned at baseline, short- and long-term follow-up (median 10 years). A gastrointestinal radiologist, blinded to clinical data, measured active MRI fistula volume on T2-weighted axial sequences at each timepoint using ITK-Snap. The primary outcome was the association between volume and pfCD Class. Secondary outcomes included identification of volume thresholds discriminating clinical state/class via ROC analysis.

**Results:**

Of 51 included patients (mean age 34.5), the majority had complex fistulae (92.1%) and 71% patients were TOpClass 2b, with 35% changing class during follow-up. MRI fistula volume measurement was feasible (median acquisition time 207 seconds, IQR 116-250). Volume was associated with disease severity, increasing across TOpClass strata (*P* < .001). ROC-derived volume thresholds effectively differentiated classes (AUROC 0.69-0.80). A ≥ 27% volume reduction was associated with clinical improvement (AUROC 0.78; sensitivity 64%, specificity 84%).

**Conclusions:**

MRI fistula volume is associated with pfCD class and disease trajectory. Volume thresholds are associated with classification shifts and clinical response, supporting their potential as objective quantitative tools. Prospective multicenter validation is warranted.

## 1. Introduction

Perianal fistulizing Crohn’s disease (pfCD) is a debilitating phenotype of Crohn’s disease (CD) that affects approximately one in 5 CD patients and is an independent predictor of long-term adverse outcomes.^1^^,^^2^ Shared decision-making between patients and clinicians can lead to timely classification of patients, resulting in the delivery of early goal-directed therapy. The development of the TOpClass classification for pfCD provides a structured framework to describe disease severity and guide treatment by aligning patient goals with tailored medical/surgical treatment.^3^^,^^4^ For instance, patients in Class 1 with minimal symptoms from their fistula can be treated expectantly, Class 2a patients may be offered early reparative surgery, Class 2b patients who are unsuitable for repair may be offered symptom control +/− palliative surgery, while those in Class 2 c-ii are likely to benefit from prompt consultation regarding defunctioning. Class 3 patients with severe symptoms despite defunctioning are likely to require early proctectomy.^5^ Classifying patients relies on a nuanced, multidisciplinary team approach, including imaging, to make informed, individualized decisions.

Radiological assessment of complex fistulae is an integral component of this multi-disciplinary approach. Regular MRI surveillance is recommended for those with Class 2 to 4 disease, or when changes in clinical status warrant further investigation.^5^ The latest ECCO-ESGAR-ESP-IBUS guidelines recommend clinical examination in combination with endoscopic examination of the rectum and MRI for the reassessment of pfCD.^6^ The PISA II study demonstrated that radiological healing was associated with fewer symptoms compared to clinical healing alone and better predicted persistent fistula closure.^7^^,^^8^ However, the lack of consensus over definitions of healing has hindered meta-analysis of existing literature and led to the development of the TOpClass criteria for radiological healing.^9^

While complete radiological healing represents a hard and clearly defined endpoint, radiological “improvement” lacks standardized thresholds and requires further validation. MRI fistula volume has been proposed as a novel metric for assessing pfCD activity,^10^^,^^11^ offering a more dynamic, 3-dimensional reflection of disease activity compared to traditional anatomical-based activity indices such as the Van Assche Index (VAI)^12^ and MAGnetic resonance Novel Index for FIstula imaging in CD (MAGNIFI-CD).^13^ These indices have typically been validated in Class 2a patients undergoing reparative surgery or stem cell treatment and may not show significant change over time in chronic or advanced disease,^14^ especially when maximum scores are reached, or fistula anatomy remains stable. In contrast, fistula volume, measured on a continuous scale, is not affected by this ceiling effect. Changes in MRI fistula volume offer a non-invasive, quantitative, and reproducible metric that may complement clinical examination and assist in classifying and initiating early goal-directed therapy. We hypothesize that higher fistula volumes are associated with greater disease activity; however, this association has yet to be validated.

Our primary objective was to evaluate the relationship between MRI-derived fistula volume and disease activity according to the TOpClass classification in pfCD. Secondary aims were to identify fistula volume thresholds that discriminate between different classes of disease activity according to the TOpClass classification and fistula drainage assessment (FDA) and to compare temporal changes in MRI fistula volume and the VAI with corresponding changes in TOpClass pfCD Class and FDA.

## 2. Methods

### Ethics

Ethical approval for this study was granted with R&D reference RD21/071 IRAS 308386. Confirmation of capacity and capability was provided by London North West University Healthcare NHS trust.

### Study design and setting

This was a retrospective observational cohort study conducted at a tertiary referral center specializing in bowel pathology. Consecutive adult patients with pfCD who underwent pelvic MRI in 2010 were identified from a locally maintained database. Baseline and follow-up MRIs within a 10-year period were retrieved from the institutional picture archiving and communication system (PACS).

### Participants

Consecutive patients were identified from a locally maintained database of pfCD patients who underwent MRI scans in 2010 and met the following inclusion criteria:

- Adults (>18 years old) with pfCD.
- Availability of 10-year follow-up clinical, endoscopic, and radiological data, including pfCD TOpClass classification at various time points.
- Minimum of one baseline MRI scan and at least one further MRI scan, >6 months apart.
- MRI scan and clinical assessment timepoints must have occurred within a 3-month interval.

Patients were excluded if they had any of the following*:*

- Non-fistulizing perianal Crohn’s disease.
- Pouch-related fistulae.
- Ano-vaginal fistulae.
- Class 4 perianal disease (ie, post-proctectomy)

### Data source and extraction

Clinical, endoscopic, histological, and radiological data were extracted from electronic patient records. Data extraction was conducted independently by 2 clinicians (E.A. and S.A.), each with over 10 years’ experience.

### Variables

Exposure variables were measured at baseline, follow-up, and long-term review (10 years). These included MRI fistula volume (active fistula tract) and the VAI. Each patient was retrospectively assigned a pfCD classification using established TOpClass criteria (Appendix A—see online supplementary material). Classifications were independently assigned by 2 researchers (E.A. and S.A.) at baseline and follow-up, with consensus reached through discussion with senior authors (A.H. and P.T.), where required. Finally, FDA was performed at each time point: an active fistula was defined as drainage on gentle finger compression of any external opening; clinical improvement was defined as a reduction in the number of draining fistulae compared to baseline; and clinical remission as the complete absence of drainage from all external openings.

### MRI image acquisition

The MRI protocol included sagittal T2-weighted and T2 fat-saturated sequences, as well as coronal-oblique and axial-oblique T2 fat-saturated sequences. Coronal and axial planes were angulated parallel and perpendicular to the anal canal. Digital imaging and communications in medicine images of the sequences were retrieved from PACS and anonymized. Only T2 fat-saturated axial sequences were required for segmentation, although access to the remaining sequences was available if required.

### MRI evaluation: data measurement and reliability

Perianal fistulae were defined as abnormal tubular connections between the enteric lumen (rectum/anal canal) and the skin. The borders of the fistula tract were defined by the transition between T2 high signal (signal brightness) and low signal fibrosis/fat on fat-suppressed sequences. All contents within the borders of the fistula were considered part of the active fistula volume. Inflammatory masses or abscesses were included in the fistula volume if considered part of the active fistula on MRI.

Previous inter-rater reliability of this method of fistula volume assessment has already been demonstrated at our institution.^10^ Baseline, short-term, and long-term follow-up anonymized MRI scans were scored by an experienced GI radiologist (P.L.) with 15 years’ experience who was blinded to clinical and demographic data throughout. Each scan was independently assessed for fistula anatomy and characteristics according to the VAI. Baseline and follow-up scans were presented as sets of images, with the reader blinded to any intervening treatment or change in disease state. Fistula volume was segmented manually using ITK-snap (Version 4.2.0), a validated open-source segmentation software.

### Outcomes

The primary outcome was the association between MRI fistula volume and pfCD TOpClass classification, including correlation of absolute and percentage volume changes with pfCD class.

Secondary outcomes included the identification of absolute and threshold MRI volume values discriminating between pfCD classes and FDA clinical states and to compare temporal changes in MRI fistula volume and the VAI with corresponding changes in TOpClass pfCD class progression and FDA.

### Bias and confounding

Patient selection bias was minimized by including consecutive eligible patients. An expert radiologist (P.L.), blinded to clinical details, measured fistula volume at each timepoint to minimize measurement bias and previous inter-rater reliability analysis confirmed the feasibility of both volumetric analysis and MRI based activity index scoring. Time intervals between MRI scans and clinical assessments were controlled (<3 months) to minimize temporal bias.

### Study size

All eligible patients from 2010 were included. No formal sample size calculation was performed, consistent with the exploratory, retrospective nature of the study.

### Statistical analysis

All analyses were performed according to a pre-specified analysis plan using IBM SPSS Statistics (Version 29). Data distribution was assessed to determine normality, with non-parametric methods applied where appropriate. Accordingly, averages are presented as median (IQR). Group differences in MRI scores across pfCD classes (1-4) and response trajectories (class down/improved, unchanged, class up/worsened) were evaluated using the Kruskal–Wallis test. ROC curves were generated to assess the diagnostic performance of MRI fistula volume using pfCD classification or FDA status as the outcome, with patients categorized as non-responders or improved (including remission). The median (IQR) time to generate MRI volume measurements was also calculated. All tests were 2-tailed, with *P* <.05 considered statistically significant.

## 3. Results

### Patient and disease characteristics

A total of 51 patients with pfCD were included in this study (Appendix B—see online supplementary material). The mean age was 34.5 years (SD 12.8), with 61% female (*n* = 31). The mean weight was 71.1 kg (SD 19). The median age at CD diagnosis was 21 years (IQR: 16.5-27), and the median disease duration was 8 years (IQR: 4-15). Fistulizing disease had been present for a median of 4 years (IQR: 2-10). On MRI, the majority of fistulae were described, using Parks classification, as transsphincteric (30/51, 58.8%), with 47/51 (92.1%) classified as complex according to American Gastroenterological Association criteria. Using the TOpClass classification, most patients were initially in Class 2b, that is, chronic symptoms not suitable for repair (36/51, 70.6%) (Appendix C—see online supplementary material). At short-term follow-up, 18/51 (35%) had changed class (13 had improvement, 5 had worsened). The median short-term follow-up was 15 months (IQR 13-31); median long-term follow-up was 120 months (96-144).

### MRI fistula volume is associated with disease activity according to the TOpClass pfCD classification

**Figure 1.**
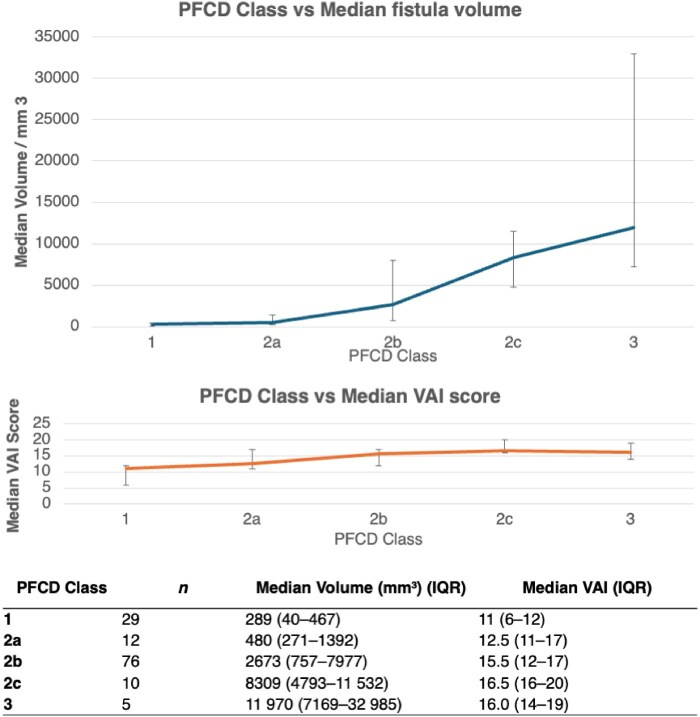
Comparison of MRI fistula volume and imaging indices across pfCD class. MRI fistula volume increases with higher pfCD class and distinguishes higher classes better than the Van Assche Index (VAI). Complete comparisons in Supplementary Material (see online supplementary material). Data are shown for baseline, short-term follow-up (median 15 months, IQR 13-31), and long-term follow-up (median 120 months, IQR 96-144), including absolute median (IQR) fistula volumes and VAI scores, stratified by PFCD class (1-3). Classes 2c-I and 2c-II were combined due to low numbers and differentiation challenges.

**Figure 2.**
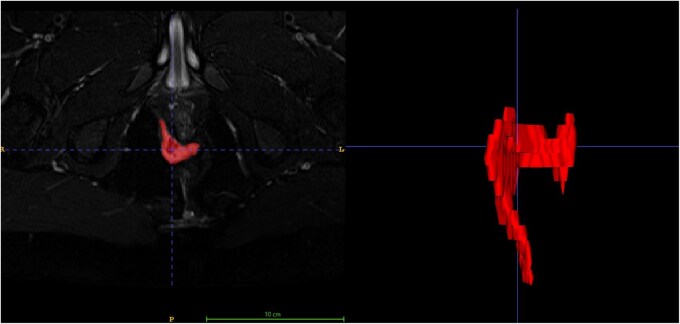
3D segmentation of posterior horseshoe using ITK-Snap. Left: Axial T2-weighted fat-saturated sequence demonstrating a posterior horseshoe abscess in a patient with complex pfCD. Right: Three-dimensional fistula reconstruction, shown from an anterior-posterior perspective of the same scan shown left generated in ITK-Snap following manual segmentation by a gastrointestinal radiologist.

MRI fistula volume (of the active fistula tract) increased with increasing disease severity according to the TOpClass classification (*P* < .001) (Figure 1).^4^ Median volume ranged from 289 mm^3^ (IQR: 40-467) in Class 1 (minimal symptoms) to 11,970 mm^3^ (IQR: 7,169-32,985) in Class 3 (severe symptoms). The median time required to calculate MRI volume was 207 seconds (IQR: 116-250). An example of a 3D segmentation model from a fistula with posterior horseshoe sepsis is shown in Figure 2.

Fistula volume continued to increase across pfCD classes without a ceiling effect, with significant differences observed between Class 1 and higher classes (2b − 3) and Class 2a and Classes 2b − 3 (all adjusted *P* < .05). In contrast, the Van Assche Index plateaued above class 2b: post-hoc comparisons showed significance only against Class 1, with no differences between Class 2a and more severe classes, indicating limited discriminatory power beyond early disease.

### MRI fistula volume thresholds can distinguish between TOpClass pfCD classification

**Figure 3.**
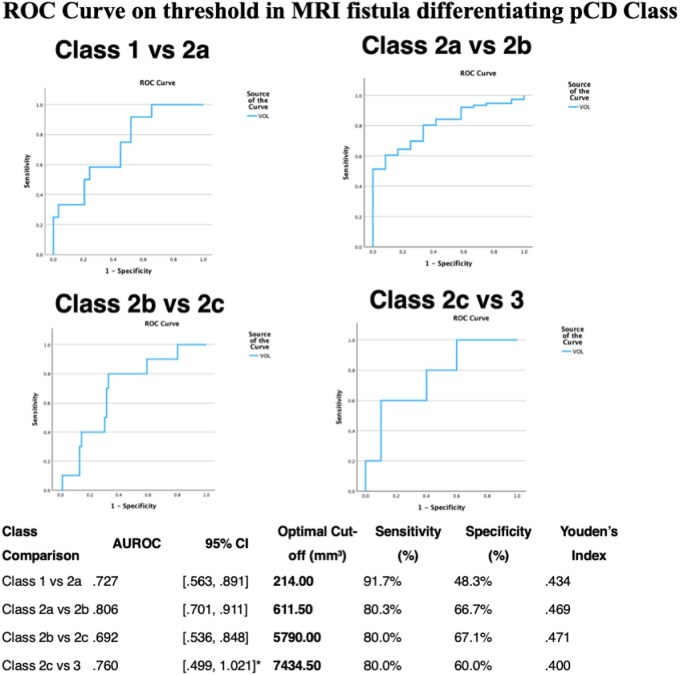
ROC Analysis of fistula volume thresholds in determining pfCD class. AUROC values range from 0.69 to 0.80 indicating good to excellent discriminatory ability of MRI fistula volume thresholds to distinguish between adjacent classes of pfCD (pfCD). The optimal cut-off volumes were selected based on the highest Youden’s Index, balancing sensitivity and specificity. **Note**. AUROC = Area Under the Receiver Operating Characteristic Curve. CI = Confidence Interval. *Upper CI bound exceeds 1.0 due to sampling variability and should be interpreted cautiously.

Receiver operating characteristic (ROC) analysis **(**Figure 3**)** identified optimal fistula volume cut-offs with good discriminatory ability to distinguish between adjacent classes. A cut-off of 214 mm^3^ differentiated Class 1 from 2a (AUROC = 0.73; sensitivity 92%, specificity 48%); 611.5 mm^3^ differentiated Class 2a from 2b (AUROC = 0.80; sensitivity 80%, specificity 67%), 5790 mm^3^ differentiated Class 2b from 2c (AUROC = 0.69; sensitivity 80%, specificity 67%), 7435 mm differentiated Class 2c vs 3 (AUROC = 0.760; sensitivity 80%, specificity 60%).

### Temporal changes in MRI fistula volume are associated with changes in TOpClass pfCD classification

Per-patient percentage change in MRI fistula volume from baseline was significantly associated with a change in pfCD class (*P* = .001, df 2) **(**Table 1**)**. Patients with ***improved*** pfCD Class had a median 65% percentage volume reduction; those ***unchanged*** had a median percentage change of -7% and those with a ***worsened*** pfCD class had a median increase in fistula volume of 69%. By comparison, median change in VAI was 0 in the ***improved*** group and in the ***worsened*** group. Over the 10-year follow-up, VAI remained unchanged in ∼ 50% cases despite TOpClass shifts, whereas MRI fistula volume reflected improvement in 18/21 (86%) cases and worsening in 7/11 (64%) cases, suggesting MRI fistula volume appears more responsive to pfCD Class shifts than existing MRI-based indices.

**Table 1.** Follow-up MRI fistula volume and MRI indices stratified by TOpClass PfCD class outcome (improved, unchanged, worsened).

PfCD class change	Metric	% Change median score (IQR) from baseline
**Improved (*n* = 21)**	*Volume (mm^3^)*	*−65 (−80 to −27)*
	VAI	0 (0 to 15)
**Unchanged (*n* = 49)**	*Volume (mm^3^)*	*−7 (−55 to + 54)*
	VAI	0 (0 to 0)
**Worsened (*n* = 11)**	*Volume (mm^3^)*	*+69 (−11 to + 218)*
	VAI	0 (−24 to 0)

From left to right: pfCD Class Change, Metric (Volume, VAI), % Change in score from baseline to follow-up.

Median (IQR) fistula volumes and median (IQR) VAI scores were calculated at paired timepoints (baseline to short-term follow-up and short-term to long-term follow-up) and stratified according to TOpClass pfCD class trajectory (**improved, unchanged, or worsened**).

For improved cases: Fistula volume decreased in 18/21 (86%), the VAI remained unchanged in 11/21 (52%).

For worsened cases: Fistula volume increased in 7/11 (64%); the VAI was unchanged in 6/11 (55%).

ROC Curve analysis was used to identify optimal MRI fistula volume thresholds to distinguish pfCD class changes (improvement or worsening) (Appendix D—see online supplementary material). A 14 % volume reduction was associated with ***class improvement*** with an acceptable level of discrimination (AUC 0.72; Sensitivity 86 %; Specificity 58 %), compared with a 0.5-point VAI reduction (AUC 0.604; sensitivity 38 %; specificity 83 %). A 27 % volume increase was the optimal threshold associated with ***class worsening*** (AUC 0.755; sensitivity 77 %; specificity 64 %), performing comparably to VAI.

### MRI fistula volume is associated with the fistula drainage assessment

MRI fistula volume was significantly associated with FDA outcomes (*P* < .001) **(**Table 2**)**. Patients in clinical remission had the lowest median fistula volume (232 mm^3^), while non-responders (ie treatment-refractory pfCD) had the highest volume (2739 mm^3^). Median VAI scores showed similar trends across response groups. Volume appeared most sensitive to change (*P* = .004): non-responders had a median change from baseline of −5 %, the improvement group −54 % (−30 to −73 %), and remission −18 % (−23 % to 93 %). By contrast, median changes in VAI were 0 across all groups, consistent with pfCD class shift analyses (Appendix E—see online supplementary material).

**Table 2.** Comparison of MRI fistula volume and VAI scores across FDA response groups (non-responder, improved, remission).

Measure	FDA	Median (IQR)	Kruskal–Wallis *P (df 2)*	Pairwise comparison	Adjusted *P*
**Volume (mm^3^)**	Non-responder (N)	2739 (721-9396)	<.001	N vs I	.141
	Improved (I)	1396 (340-4270)		N vs R	<.001
	Remission (R)	232 (0-649)		I vs R	.011
**VAI**	Non-responder (N)	16 (12-19)	<.001	N vs I	.787
	Improved (I)	14 (11-17)		N vs R	<.001
	Remission (R)	8.5 (0-12)		I vs R	<.001

Median (IQR) values for MRI fistula volume and Van Assche index scores were stratified by paired FDA outcomes (Non-responder (N), Improved (I), Remission (R)). Pairwise comparisons were conducted using the Kruskal–Wallis test between Non-responder vs Improved, Non-responder vs Remission, and Improved vs. Remission strata. Corresponding *P*-values are reported in the rightmost column. Volume and VAI performed comparably in assessment of FDA, which is a structural-based outcome, ie, the fistula tract has closed in remission and therefore structural MRI-based indices are more likely to reflect this.

**Figure 4.**
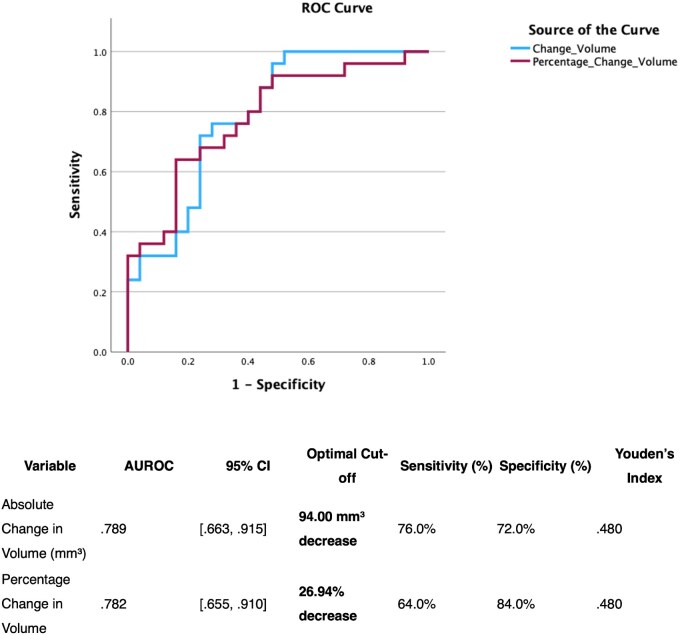
ROC curve metrics for determining short-term clinical improvement based on MRI fistula volume changes (*n* = 51). The highest AUC value of 0.79 was obtained by using a threshold of a reduction in volume of 94 mm3 or 27% (AUC 0.78) to determine clinical improvement according to the FDA. Receiver operating characteristic (ROC) curve analysis was performed to determine optimal cut-off values for absolute and percentage reduction in fistula volume in distinguishing between FDA status, as well as absolute reduction Van Assche index. The optimal reduction in VAI was 0.5 points with a lower AUC of 0.66 (sensitivity 34%, specificity 90%). The area under the curve (AUC) with 95% confidence intervals is reported, with sensitivity, specificity, and Youden’s index values presented in the final 3 columns.

ROC curve analysis **(**Figure 4**)** identified optimal thresholds for absolute and percentage MRI fistula volume changes associated with clinical improvement (FDA). An optimal cut-off of 27% volume reduction was identified (AUROC 0.78 [95% CI 0.66-0.91]; sensitivity 64%, specificity 84%) indicating a moderate level of discrimination.

## 4. Discussion

### Key findings and interpretation

This study demonstrates that MRI-derived fistula volume, both as an absolute measure and as a dynamic variable, is a promising radiological biomarker for assessing disease activity in pfCD. We found that baseline fistula volume is strongly associated with disease severity as stratified by the TOpClass classification system and that changes in volume over time closely mirror clinical and radiological trajectories. Baseline MRI fistula volume increased stepwise across ascending TOpClass categories, with statistically significant differences (*P* < .001) between each class. Median volumes ranged from 289 mm^3^ in Class 1 to nearly 12,000 mm^3^ in Class 3 (before proctectomy), suggesting that volumetric burden reflects disease burden according to the new TOpClass classification. ROC analyses identified clinically relevant thresholds, with AUC values of 0.692-0.806, indicating that fistula volume has moderate ability to discriminate between adjacent classes. For example, a threshold of 612 mm^3^ differentiated Class 2a from 2b with an AUROC of 0.806, 80.3% sensitivity, and 66.7% specificity. Whether this threshold predicts successful repair requires further study.

Importantly, dynamic changes in volume corresponded closely to TOpClass trajectory. Patients who improved (class down) demonstrated a median volume reduction 65% from baseline. Those who remained unchanged had minimal volume reduction (median change −7%), while patients who worsened (class up) showed a median 69% increase in fistula volume. MRI volume change was also associated with short-term clinical improvement: a ≥27% volume reduction from baseline was associated with clinical improvement (AUROC = 0.78), with reasonable discriminatory performance. These findings provide strong support for MRI fistula volume as a dynamic and objective surrogate of both clinical and radiological disease activity in pfCD. This aligns with prior studies suggesting that volume is a sensitive marker of therapeutic response,^10^^,^^11^ but our study uniquely validates this within the patient-orientated TOpClass framework.

In our cohort, the VAI showed little change in advanced disease, reflected by a median change of 0 even among patients with clinically meaningful improvement. These ordinal indices are limited by ceiling effects, so patients with highly complex fistulae may appear “unchanged” despite substantial anatomical progression or improvement, making them less sensitive to incremental improvements. By contrast, MRI fistula volume may offer greater responsiveness by capturing reductions in complexity without requiring overt anatomical change. Volume is continuous, highly sensitive to change (only one pair of readings was identical from baseline to follow-up), and captures both improvement and deterioration across the disease spectrum. Consequently, volume identifies changes often missed by MRI indices and serves as a dynamic, objective surrogate of clinical and radiological activity within the TOpClass framework. Limitations include reduced sensitivity for very small tracts, but reproducibility is excellent (ICC 0.99, 95 % CI 0.97-0.99),^10^ and AI-based volumetry could further overcome ceiling and responsiveness limitations for both clinical assessment and trial endpoints.

### Existing evidence

Current MRI-based indices such as the VAI and the more recent MAGNIFI-CD offer structured assessment of perianal disease but have limitations: they are partly subjective, some require contrast, and may not adequately reflect the fluctuating and dynamic course of fistula healing or progression. Furthermore, many existing indices including MAGNIFI-CD do not align well with validated indices including Perianal Disease Activity Index (PDAI) and IBDQ.^13^ However, patients with pfCD consistently emphasize the importance of scan findings in understanding and assessing their disease, often viewing imaging results as a key indicator of progress or deterioration.^15^ The most widely used MRI based activity index, MAGNIFI-CD, was developed prior to the new TOpClass classification ­system.^3^^,^^4^ Attempts to date at radiological validation of the TOpClass classification have suggested that baseline MRI indices did not appear to correlate with changes in classification over time.^16^ In our cohort, median changes in the Van Assche Index (VAI) remained at 0 throughout, highlighting the limitations of relying on a single index with reduced sensitivity in both very simple and very complex fistula presentations. Likewise, median VAI scores were similar across classes 2b, 2c, and 3, indicating the ceiling effect.

### Strengths and limitations

This is a large longitudinal imaging dataset assessing fistula volume in pfCD, with paired clinical, radiological, and classification data and a median follow-up duration of 10 years. Use of the TOpClass classification allows for more granular tracking of disease trajectory, and our volume analysis was performed blinded and independently, enhancing validity.

This study has several limitations that warrant consideration. First, its retrospective, single-center design may limit generalizability, particularly as patients at tertiary IBD centres tend to have more complex disease and receive more intensive imaging and intervention than the broader Crohn’s population. However, the longitudinal nature of the study and comparison with existing MRI indices help mitigate some of the constraints of a single-center dataset. This retrospective study is also limited by our inability to control for confounding treatment effects including medications, setons, and surgery which were heterogeneous in their use. Future multi-center, prospective studies should focus on the relationship between fistula volume and specific therapeutic interventions/different classes of patients.

The distribution of patients across TOpClass categories was also uneven, with small numbers in classes 2a, 2c, and 3 reducing statistical power and limiting the robustness of cut-off thresholds derived from ROC analysis. Although some patients contributed multiple MRI assessments, our analyses focused on independent volumetric measurements per scan. While statistical adjustments were applied where relevant, residual within-patient correlation may still influence the results. Moreover, the retrospective nature of the study and the relatively recent introduction of the TOpClass classification meant that classes were not prospectively defined. Clinical researchers were not blinded to follow-up history and disease evolution, introducing potential bias. This was mitigated to some degree by applying recently published, standardized multi-center criteria for assigning pfCD classes retrospectively.^3^

Fistula volume measurements relied on manual segmentation by a single trained reader and in non-expert hands manual segmentation may be initially time consuming and subject to variability, particularly in anatomically complex or post-operative cases. Nevertheless, our previous feasibility study demonstrated that volume measurement is practical, reliable, and reproducible, with excellent interobserver agreement even among raters with varying MRI experience.^10^ Post-processing of a single MRI fistula scan takes around 5 minutes per scan, a limitation that could substantially be reduced with dedicated software and the development of AI.

Imaging heterogeneity over the study period, including differences in scanner types and minor protocol variations, may also have influenced volume estimates. A further limitation is that the lack of contrast-enhanced MRI precluded calculation of the full MAGNIFI-CD score. Consequently, we relied on the Van Assche Index which does not require contrast. The primary focus of our study was on shifts in pfCD class and clinical status over time, although future studies should aim to perform a direct comparison using the full contrast-dependent MAGNIFI-CD. Additionally, our outcomes focused on radiological and clinician-reported endpoints, without incorporating validated patient-reported outcomes such as the Crohn’s Anal Fistula Quality of life (CAF-QOL) scale,^17^ which was developed after the study inclusion period or PDAI.^18^ As such, the relationship between volume changes and meaningful improvements in quality of life remains unclear.

### Clinical and research implications

Volume offers a continuous, quantifiable, and intuitive metric that correlates with disease severity and reflects meaningful change over time. As an objective biomarker, volume change may be easier to interpret than composite scores or radiologist-derived impressions, providing clinicians with a tangible tool to track progress and communicate treatment response to patients in simple terms: whether their fistula is improving, unchanged, or worsening. While class changes can often be assessed clinically, volumetry may detect subtle or early changes in fistula burden not apparent on routine examination, offering a potential tool for monitoring treatment response and informing future predictive models. A clinician could, for example, tell a patient that their fistula has reduced in volume by 27% (for example), reflecting meaningful radiological improvement, even in the absence of complete healing. Conversely, a volume increase may signal early deterioration, prompting timely escalation or surgical referral. Where MRI activity indices such as VAI and MAGNIFI-CD are used, the addition of volume measurements as an adjunct can help mitigate some of the limitations of lack of change over time and the ceiling effect.

Volume also enhances the ability to stratify disease severity. It aligns well with the TOpClass framework, enabling classification of patients into clinically actionable categories. Low volumes (<214 mm^3^) could help identify patients in early disease (Class 2a), where patients may be suitable for fistula closure, while high volumes (>7,400 mm^3^) may aid in identifying patients with complex, refractory disease (2c/Class 3), where counselling about surgical options including defunctioning -ostomy and proctectomy may be appropriate. These cut-offs, though requiring external validation, are clinically plausible and offer a reproducible method for staging and prognostication.

For researchers, fistula volume offers a scalable and potentially automatable outcome measure that aligns with both short- and long-term endpoints, including radiological improvement.^9^ Importantly, our findings support volume-based improvement as a meaningful outcome distinct from complete healing, enabling clinical trials to capture partial responses rather than relying solely on binary healed/not-healed classifications. Integrating volumetric measures into existing MRI activity scores could combine the responsiveness and continuous nature of volume with the anatomical detail of established indices, providing a more comprehensive and sensitive assessment of treatment benefit.

Further work in larger prospective studies is needed to determine whether volume-based measures also have predictive value, both pre-intervention in identifying patients most likely to benefit and post-intervention in estimating sustained success. Future research should also assess the association of signal intensity with disease severity, correlations between volume change and fistula healing or repair, quality of life, ostomy or proctectomy rates, and the development of Class 4 disease.

### Conclusion

The binary TOpClass definitions of radiological improvement and healing aim to standardize MRI-based outcomes in clinical trials and research, yet current evidence lacks a robust quantitative imaging correlate. Our findings help address this gap by demonstrating that MRI fistula volume, both as an absolute value and as a dynamic measure of change correlates closely with TOpClass classification and tracks clinically meaningful improvement and worsening over time. Volume reduction offers a sensitive, clinically relevant, and quantifiable indicator of therapeutic response, providing achievable radiological endpoints such as radiological improvement. Importantly, the absence of a ceiling effect with fistula volume allows progressive disease burden to be quantified, overcoming a key limitation of existing MRI-based indices. Larger prospective, multi-center, multi-reader studies are now warranted to confirm inter-rater reliability, assess external validity, and examine correlations with quality of life and patient-reported outcomes. Future work should focus on the development of AI-based tools for rapid, reproducible segmentation, and on integrating volumetric metrics into longitudinal registries and real-world data platforms. Prediction of outcome would be a key benefit of volume assessment.

## Supplementary Material

jjaf216_Supplementary_Data

## Patient and public involvement

No patients were directly involved in this study.
