# Supplementary material for: Real-world evaluation of MRI fistula volume as a radiological biomarker of disease activity in perianal fistulizing Crohn’s disease
Source: J Crohns Colitis. 2025 Nov 29;19(11):jjaf216. doi: 10.1093/ecco-jcc/jjaf216 (PMC12715302; doi:10.1093/ecco-jcc/jjaf216)
Supplement: jjaf216_Supplementary_Data [file jjaf216_supplementary_data.zip › V2 Supplementary material MRI Fistula Volume 29.10.2025.docx]

**Appendix A: TOpClass scoring criteria** (Adapted from Anandabaskaran et al, 2025 (19))

| **TOpClass Classification** | **TOpClass Definition** | **Study Transition Points** |
| --- | --- | --- |
| Class 1 | Minimal symptoms and anorectal disease burden, requiring minimal intervention over time | - History of perianal fistula on clinical examination or MRI without symptoms, no surgical or medical therapy offered for perianal symptoms - Clinical remission of in a previously symptomatic patient for at least 12 months |
| Class 2a | Symptomatic fistulae suitable for combined medical and surgical closure or repair (including seton removal) and patient goal is fistula closure | - Symptomatic perianal fistula including drainage of abscess for which definitive surgical closure or seton removal was offered |
| Class 2b | Chronic symptoms related to fistulae (pain and discharge) that aﬀect quality of life. Fistulae are currently unsuitable for surgical  repair, and patient goal is symptom control | - Symptomatic perianal fistula including drainage of abscess for which surgical closure was not offered or seton removal was not attempted - Symptomatic perianal fistula for which patient declined surgical closure or seton removal - Newly symptomatic fistula for which seton was inserted and/or medical therapy was initiated |
| Class 2c-i | Early and rapidly progressive  disease destructive to the  perineum or to quality of life  (or both), such that early  intervention with  defunctioning ostomy and  sometimes early proctectomy  is required | - Rapid progression of symptoms over 3-6 months needing ongoing medical and/or surgical treatment for symptom control - Surgical closure or seton removal not offered in the same 3-6 month period - Defunctioning offered or undertaken |
| Class 2c-ii | Gradually debilitating  symptomatic fistulae  unsuitable for surgical repair,  which cause severe symptoms,  limiting quality of life so  markedly that defunctioning  ostomy is required to restore  quality of life. Patient goal is  symptom control | - Gradually worsening symptoms over 12 months or longer - Surgical closure or seton removal not offered - Defunctioning offered or undertaken |
| Class 3 | Severely symptomatic disease  (despite defunctioning),  with irreversible perineal  destruction, or symptoms  limiting quality of life so  markedly that proctectomy  is required | - Ongoing symptoms despite defunctioning - Proctectomy discussed or undertaken |
| Class 4a | Symptomatic sinuses or  wounds suitable for  combined medical and  surgical closure or repair and  patient goal is sinus closure | - Symptomatic sinus following proctectomy - Surgical closure discussed or attempted |
| Class 4b | Chronic symptoms related to  sinuses or wounds that aﬀect  quality of life and that are  unsuitable for surgical repair,  or patient goal is symptom  control | - Symptomatic sinus following proctectomy - Surgical closure not offered or declined by patient |

**Appendix B: Baseline characteristics of study cohort (n = 51)**

| **Characteristic** | **n (%) / Median (IQR)** | **Mean (SD)** | **Range** |
| --- | --- | --- | --- |
| **Demographics** |  |  |  |
| Age (years) | — | 34.5 (12.84) | 17–85 |
| Weight (kg) | — | 71.1 (18.57) | 46–152 |
| Sex |  |  |  |
| Female | 31 (61%) | — | — |
| Male | 20 (39%) | — | — |
| Smoking status |  |  |  |
| Non-smoker | 34 (67%) | — | — |
| Smoker | 9 (18%) | — | — |
| Ex-smoker | 8 (16%) | — | — |
| Age at Crohn’s diagnosis (years) | 21 (16.5–27) | — | 11–60 |
| Duration of Crohn’s disease (years) | 8 (4–15) | — | 0–38 |
| Duration of Crohn’s fistula (years) | 4 (2–10) | — | 0–22 |
| **Medication at baseline** |  |  |  |
| Infliximab | 23 (45%) | — | — |
| Adalimumab | 7 (14%) | — | — |
| Azathioprine | 8 (16%) | — | — |
| Mesalazine | 4 (8%) | — | — |
| Mercaptopurine | 1 (2%) | — | — |
| Nil | 4 (8%) | — | — |
| **Previous surgery** |  |  |  |
| Seton insertion | 33 (65%) | — | — |
| Wide drainage | 7 (14%) | — | — |
| Advancement flap | 1 (2%) | — | — |
| Right colectomy | 6 (12%) | — | — |
| Subtotal colectomy | 5 (10%) | — | — |
| Defunctioning ostomy | 5 (10%) | — | — |
| Small bowel resection | 3 (6%) | — | — |
| Nil | 4 (8%) | — | — |
| **Fistula characteristics** |  |  |  |
| *Parks Classification* |  |  |  |
| Intersphincteric | 15 (29.4%) | — | — |
| Transsphincteric | 30 (58.8%) | — | — |
| Extrasphincteric | 2 (3.9%) | — | — |
| Suprasphincteric | 4 (7.8%) | — | — |
| Horseshoe Extension | 16 (31%) | — | — |
| Supralevator Extension | 10 (19.6%) | — | — |
| New Track Present | 10 (19.6%) | — | — |
| Cavity Present | 17 (33.3%) | — | — |
| Simple Fistula | 4 (7.8%) | — | — |
| Complex Fistula | 47 |  |  |

**Appendix C: Stratification of patients into PFCD Class**

| **pfCD Class** | **Baseline n (%)**  **(Total n = 51)** | **Short term Follow Up n (%) (Total n = 51)** | **Long-term Follow up n (%) (Total n = 43)** |
| --- | --- | --- | --- |
| 1 | 8 (15.7%) | 12 (23.5 %) | 12 (27.9 %) |
| 2a | 2 (3.9 %) | 5 (9.8 %) | 6 (11.8 %) |
| 2b | 36 (70.6 %) | 28 (50.9 %) | 20 (46.5 %) |
| 2c-i | 1 (1.8 %) | 1 (1.8 %) | 0 (0%) |
| 2c-ii | 3 (5.9 %) | 4 (7.8 %) | 6 (13.9 %) |
| 3 | 1 (1.8 %) | 1 (1.8 %) | 2 (4.7 %) |
| 4a | 0 (0%) | 0 (0%) | 1 (2.3 %) |
| 4b | 0 (0%) | 0 (0%) | 3 (7.0 %) |

*Perianal fistulising Crohn’s disease (pfCD) class was assigned independently by two researchers, with discrepancies resolved by consensus. Classification was undertaken at baseline, short-term follow-up (median 15 months, IQR 13 – 31 months), and long-term follow-up (median 120 months, IQR 96 – 144 months).*

**Stratification of patients into Class Improved, Unchanged or Worsened**

|  | **Baseline**  **(n = 51)** | **Baseline 🡪 Short term**  **(n = 51)** | **Short-term 🡪Long Term**  **(n = 43)** |
| --- | --- | --- | --- |
| Improved (Class down) | N/A | 13 (25.5 %) | 10 (23.3 %) |
| Unchanged (Class same) | N/A | 33 (64.7 %) | 20 (46.5 %) |
| Worse (Class up) | N/A | 5 (9.8%) | 13 (30.2 %) |
| Median time to follow up / months (IQR) |  | 15 (13 – 31) | 120 (96 – 144) |

*Presents inter-interval class transitions, categorised as class improved, unchanged or worse.*

| **Appendix D: ROC Curve analysis to identify thresholds in Volume, VAI and non-contrast MAGNIFI for pfCD Class shifts i.e. Improvement or Worsening class**   \| **Parameter** \| **Threshold** \| **AUC** \| **Sensitivity** \| **Specificity** \| **Youden’s Index** \| **Direction of PFCD Class Change** \| \| --- \| --- \| --- \| --- \| --- \| --- \| --- \| \| **% Volume** \| 14% reduction \| 0.720 \| 86% \| 58% \| 0.433 \| **Improvement** \| \| **Absolute Volume** \| 94 mm³ reduction \| 0.651 \| 86% \| 53% \| 0.390 \| **Improvement** \| \| **% VAI Change** \| 14% reduction \| 0.607 \| 33% \| 90% \| 0.232 \| **Improvement** \| \| **Absolute VAI** \| 0.5 point reduction \| 0.604 \| 38% \| 83% \| 0.214 \| **Improvement** \| \| **% Volume** \| 27% increase \| 0.755 \| 77% \| 64% \| 0.404 \| **Worsening** \| \| **Absolute Volume** \| 1301 mm³ increase \| 0.674 \| 87% \| 45% \| 0.326 \| **Worsening** \| \| **% VAI** \| 2.5% increase \| 0.773 \| 94% \| 45% \| 0.397 \| **Worsening** \| \| **Absolute VAI** \| 3 point increase \| 0.773 \| 99% \| 36% \| 0.349 \| **Worsening** \|   *Receiver operating characteristic (ROC) curve analysis with Youden’s index was used to determine optimal cut-off values for predicting binary pfCD class changes (improved or worsened). The upper section of the table presents optimal thresholds for reduction in fistula volume or MRI-based indices predictive of class improvement, while the lower section presents optimal thresholds predictive of class worsening.*  **Appendix E: Association between Volume, Van Assche Index, non-contrast MAGNIFI-CD and Fistula Drainage Assessment (FDA) outcome (Non-responder (N), Improved (I), Remission (R))** |
| --- | --- | --- | --- | --- | --- | --- | --- | --- | --- | --- | --- | --- | --- | --- | --- | --- | --- | --- | --- | --- | --- | --- | --- | --- | --- | --- | --- | --- | --- | --- | --- | --- | --- | --- | --- | --- | --- | --- | --- | --- | --- | --- | --- | --- | --- | --- | --- | --- | --- | --- | --- | --- | --- | --- | --- | --- | --- | --- | --- | --- | --- | --- | --- |
|  |

| - - 1. ***% Change IN VOLUME*** | **Median % Reduction in volume (IQR)** | **Independent-Samples Kruskal-Wallis Test p value** |  |  |
| --- | --- | --- | --- | --- |
| Non-responder | - 5 % (-105% - +45) %) | **0.004** | N v I | **0.001** |
| Improved | + 54 % (-73 % - - 30 %) |  | N v R | 0.088 |
| Remission | + 18 % (-23 % - + 93 %) |  | I v R | 0.982 |
| - - 1. ***Absolute Change in VAI*** | **Median Change in VAI (IQR)** | **Independent-Samples Kruskal-Wallis Test p value** |  |  |
| Non-responder | 0 (0 – 0) | **0.002** | N v I | 0.120 |
| Improved | 0 (0 – -1) |  | N v R | **0.007** |
| Remission | 0 (0 – -6) |  | I v R | 0.740 |

Significant differences were observed between non-responders, improved, and remission groups for baseline volume, VAI, and non-contrast MAGNIFI-CD scores (all p < .001), with remission showing the lowest median values and non-responders the highest.
For absolute and percentage changes, non-responders demonstrated significantly smaller reductions in volume, VAI, and non-contrast MAGNIFI-CD compared with improved or remission groups (p ≤ .007 for key contrasts), although improvements between the improved and remission groups were generally not statistically different.
Baseline differences were most marked between non-responders and remission (p < .001 for volume, VAI, and MAGNIFI), while volume change and % change were significantly greater in improved vs non-responders (p = .001) but similar between improved and remission.
